# Reseek-Arrhythmia: Empirical Evaluation of ResNet Architecture for Detection of Arrhythmia

**DOI:** 10.3390/diagnostics13182867

**Published:** 2023-09-06

**Authors:** Shams Ul Haq, Sibghat Ullah Bazai, Ali Fatima, Shah Marjan, Jing Yang, Lip Yee Por, Mohd Anjum, Sana Shahab, Chin Soon Ku

**Affiliations:** 1 Department of Computer Engineering, Balochistan University of Information Technology, Engineering, and Management Sciences (BUITEMS), Quetta 87300, Pakistan; engr.shams562@gmail.com (S.U.H.); fatimaali21358@gmail.com (A.F.); 2 Department of Software Engineering, Balochistan University of Information Technology, Engineering, and Management Sciences (BUITEMS), Quetta 87300, Pakistan; 3 Department of Computer System and Technology, Faculty of Computer Science and Information Technology, Universiti Malaya, Kuala Lumpur 50603, Malaysia; yj741655109@163.com (J.Y.); porlip@um.edu.my (L.Y.P.); 4 Department of Computer Engineering, Aligarh Muslim University, Aligarh 202002, India; mohdanjum@zhcet.ac.in; 5 Department of Business Administration, College of Business Administration, Princess Nourah Bint Abdulrahman University, P.O. Box 84428, Riyadh 11671, Saudi Arabia; sshahab@pnu.edu.sa; 6 Department of Computer Science, Universiti Tunku Abdul Rahman, Kampar 31900, Malaysia

**Keywords:** heart disease, arrhythmia detection, ECG, convolutional neural network, deep learning, convolutional blocks, identity blocks

## Abstract

Arrhythmia is a cardiac condition characterized by an irregular heart rhythm that hinders the proper circulation of blood, posing a severe risk to individuals’ lives. Globally, arrhythmias are recognized as a significant health concern, accounting for nearly 12 percent of all deaths. As a result, there has been a growing focus on utilizing artificial intelligence for the detection and classification of abnormal heartbeats. In recent years, self-operated heartbeat detection research has gained popularity due to its cost-effectiveness and potential for expediting therapy for individuals at risk of arrhythmias. However, building an efficient automatic heartbeat monitoring approach for arrhythmia identification and classification comes with several significant challenges. These challenges include addressing issues related to data quality, determining the range for heart rate segmentation, managing data imbalance difficulties, handling intra- and inter-patient variations, distinguishing supraventricular irregular heartbeats from regular heartbeats, and ensuring model interpretability. In this study, we propose the Reseek-Arrhythmia model, which leverages deep learning techniques to automatically detect and classify heart arrhythmia diseases. The model combines different convolutional blocks and identity blocks, along with essential components such as convolution layers, batch normalization layers, and activation layers. To train and evaluate the model, we utilized the MIT-BIH and PTB datasets. Remarkably, the proposed model achieves outstanding performance with an accuracy of 99.35% and 93.50% and an acceptable loss of 0.688 and 0.2564, respectively.

## 1. Introduction

**Figure 1:**
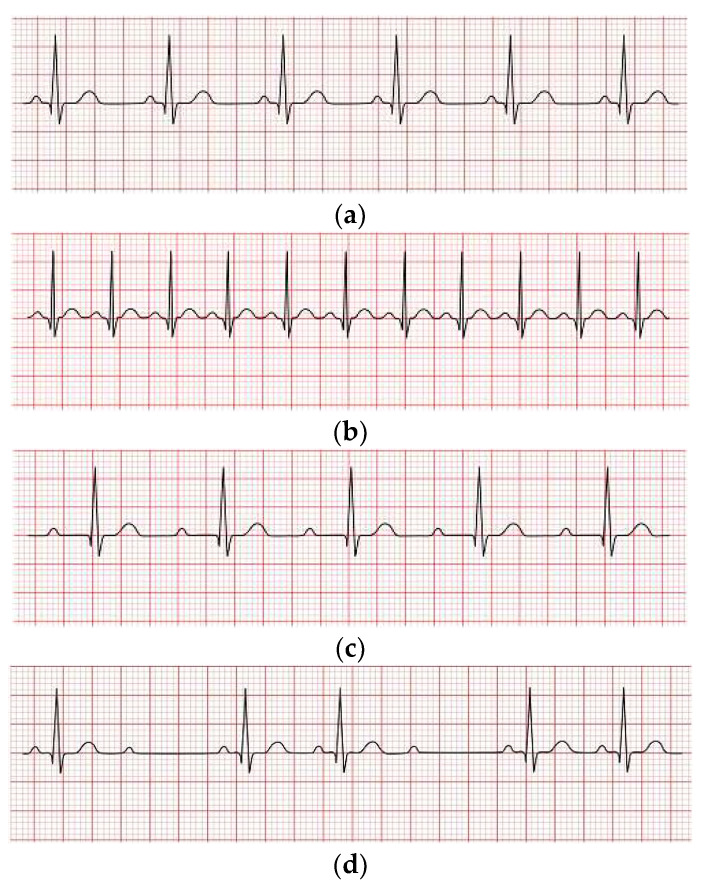
Graphical representation of a normal rhythm and different arrhythmia conditions: (**a**) Displays the normal rhythm; (**b**) Describes the tachycardia arrhythmia heartbeat; (**c**) Describes the bradycardia arrhythmia heartbeat; (**d**) Shows an irregular heartbeat.

Heart disease (HD) is a lethal condition affecting a large number of human beings around the globe when compared to other diseases. According to the World Health Organization (WHO), HD is the leading cause of death globally, and it is estimated that HD is responsible for 17.9 million deaths per year [1]. Numerous conditions can lead to HD, including excessive hypertension, uncontrolled angina, cardiac arrhythmias, heart attacks, and so on. Therefore, early and timely arrhythmia detection and classification are crucial. This study focused on the automatic detection and classification of arrhythmias. A cardiac arrhythmia occurs when electrical impulses in the heart do not work correctly, resulting in irregular heartbeats. Heartbeats can be categorized into the following four kinds: (i): normal rhythm where the heart functions properly and the heartbeat rate is about 60−100 bpm; (ii): tachycardia occurs when the heart beats too quickly (more than 100 bpm); (iii): bradycardia happens when the ordinary person’s heartbeats become slower than average (less than 60 bpm) [2]; and (iv): heart rhythm irregularity (as seen in Figure 1a–d, respectively).

**Figure 2:**
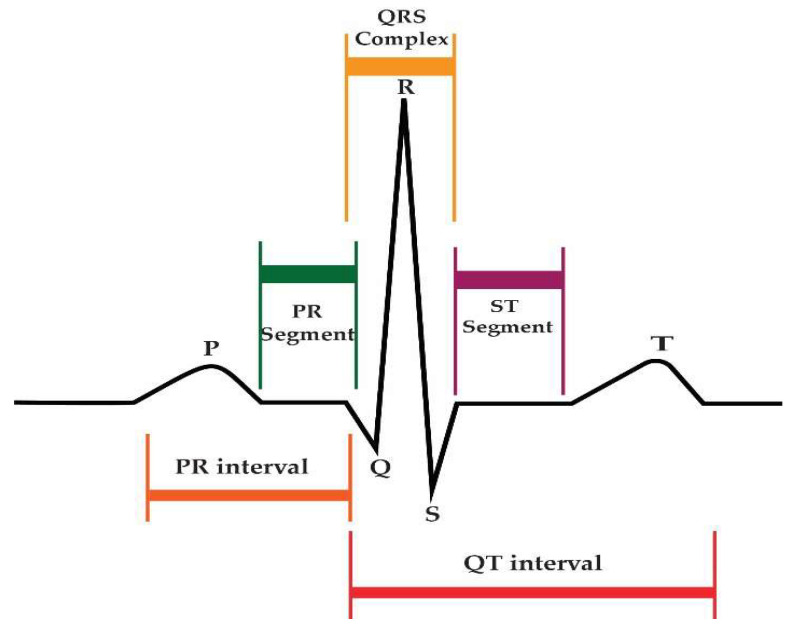
Main waves from the normal rhythm with the specifications.

Arrhythmias can be diagnosed with the help of an electrocardiogram (ECG). The ECG is a well-established tool in cardiology for monitoring a patient’s heart condition. The 12-lead ECG is the most effective equipment for recording cardiac rhythm. The leads are the recording channels, and they are lead I, lead II, lead III, aVR, aVL, aVF, V1, V2, V3, V4, V5, and V6. Every channel records from a distinct perspective. Every normal ECG heartbeat has three main waves, namely the P-wave, T-wave, and R-point, as shown in Figure 2.

Arrhythmia HD refers to abnormal heart rhythms, requiring trained professionals to visually analyze complex and diverse ECG data. This process can be time-consuming and costly. Accurate identification of arrhythmia beats is challenging due to variations in their forms and patterns. Extracting valuable insights from complex medical data records remains a significant challenge in the medical field. To overcome the challenges, several techniques are used for automatic detection and classification [3,4,5,6].

Computer vision, artificial intelligence (AI), and its subsets, machine learning (ML) and deep learning (DL), are used for automatic arrhythmia detection and classification. Recent deep learning advancements offer practical frameworks for comprehensive models with high-dimensional data. AI has gained astounding success in detecting and discriminating high-level images in recent years. In 2012, for instance, a deep learning model called AlexNet, which was composed of a convolutional neural network (CNN) [7] model, achieved 84.7 percent accuracy. The 62 million parameters of the model were optimized after being trained on 1,400,000 images. Accuracy was continuously improved by the development of new models, including VGG16, VGG19, ResNet50, Inception, and Xception. Researchers have been encouraged to use CNN-based models in the healthcare discipline for illness detection utilizing healthcare data, particularly images, because of the models’ outstanding performance [8]. 

The main contributions of this paper are as follows:

- Proposes Reseek-Arrhythmia model to detect and classify arrhythmia HD.
- Utilizes two different datasets, namely the MIT-BIH and PTB datasets, to evaluate the proposed model.
- Handles unbalanced and noisy data.
- Evaluates the proposed model using various performance metrics, including accuracy, precision, recall, F1-score, and loss.
- Compares the performance of the proposed model with previous studies in the field.

## 2. Related Work

Numerous studies have been undertaken regarding the identification and classification of arrhythmias. The purpose of [9] is to investigate the application of machine learning for automated ECG diagnosis in myocardial infarction (MI). DenseNet and CNN models were created and trained to utilize database ECG signals. DenseNet outperformed CNN in terms of classification accuracy and computational complexity. The article gives important insights into the labeling decisions of deep models for MI and healthy ECGs, which might help clinical acceptance and adoption in MI diagnosis. In another approach using the MIT-BIH datasets, four different forms of arrhythmia were identified with a 99.38 percent accuracy rate using the artificial neural network (ANN) model [10]. For a concise summary and comparison of the methods and results of the various studies discussed, refer to Table 1.

In this study, the performance of neural networks in identifying electrocardiogram (ECG) patterns as normal or abnormal is investigated. With 91.9% accuracy in binary classification and 75.7% accuracy in multi-class classification, the neural network (NN) model, including support vector machines (SVMs), random forests, and logistic regression, outperforms. When compared to CPUs, the utilization of GPUs dramatically speeds the training process. The subject of this work is cardiac dysrhythmia, which kills approximately 500,000 people in the United States each year [34].

Former research utilized the multi-layer neural network approach for multi-class and binary detection (normal or abnormal). Inverse and stochastic gradient descent were used in the approach; the greater the number of neurons employed by the researcher, the lower the accuracy. As a result, the number of neurons must be adjusted based on the data. For the conclusion, the author utilized two hidden layers with 50 nodes each. A GPU booster was utilized to shorten the learning time. As a consequence, the neural network could now function six times quicker. The accuracy on binary classes was 91.9 percent and 75.7 percent on multi-class. This method used the deep belief network (DBN) and deep learning research to detect arrhythmia. ECG data were divided into six categories, including normal data, while SVM and ANN were also used for detection and DBN for the extraction of the features. The Gaussian kernel SVM achieved the best accuracy of 95.49 percent [35]. Another study utilized deep learning to find different disorders in the heartbeat variability data of individuals. The first method was applied to labeled data using convolutional neural networks. 

The second strategy used “stacked auto-encoders” and “restricted Boltzmann” machines to develop different attributes of training. The former method had an accuracy of 88.45 percent, while the latter had an accuracy of 90.64 percent [36]. This study compared many standard detection algorithms, including the naive Bayes (NB) classifier, the SVM, the ANN, and random forests (RF). Both the SVM and RF used an arrhythmia dataset. The outcome of the 16 highest accuracies was achieved by SVM and RF, as claimed by the author, which is 77.4 percent [37]. The detection methods of ANN and SVM were compared by [38], using the positive predictive value of cardiovascular disorders as the foundation. In order to find patients with coronary artery disease, they examined patient medical information from numerous institutions. The data, which consist of 1324 occurrences and 25 attributes, were divided into different sets for the algorithm in proportions of 70% and 30%, respectively. According to their research, the SVM performs better than the ANN in terms of accuracy and performance [38]. Another article [39] suggested excessively prominent detection methods while mining data. The author used multi-layered feeding-forward-backward propagation techniques such as SVM, k-nearest neighbors, RF, decision trees (DT), NB, and ANN for their study and observed the highest accuracy of 98.15 percent.

This study describes a unique method for classifying cardiac arrhythmias based on ECG data. Using different approaches, including CNNs, the proposed method integrates several characteristics retrieved from the ECG data. Morphological, chronological, and statistical characteristics are among the features. A composite feature vector is produced and fed into a CNN classifier for label prediction. On the MIT-BIH database, the system obtains an average classification accuracy of 98% [40]. They used AI (ML and DL) to estimate the patients’ potential for acquiring cardiovascular disease. The study relied on data collected from the 2011–2013 National Inpatient Sample (NIS), which is in the United States [41]. On a publicly available UCI heart-disease dataset, the author used a deep convolutional neural network (DCNN) to distinguish between healthy and nonhealthy cases by extracting characteristics from the dataset. Accuracy, precision, recall, and the F1 measure are performance measures that verify the suggested approach, which obtains a validation accuracy of 91.7%. The experimental findings indicate the approach’s usefulness in real-world scenarios [42]. 

Prior studies examined the effectiveness of the categorization method for predicting heart disease using LR and back-propagation neural networks (BFNNs). In order to categorize cardiac disease, they compared parametric and non-parametric techniques. They obtained a private dataset, which has 270 records and 13 attributes, 11 of which are used to support their research. Using a 10-fold cross-validation method, the experimenters determined the estimation of their detection models’ objectivity. According to their findings, LR obtained 91 percent accuracy, and BFNN achieved 88 percent accuracy [43]. In [44], they examined the performance of computational intelligence systems for identifying cardiac disease. They employed seven methods of computational intelligence, including k-nearest neighbors, DT, support vector machines, random forests, deep neural networks, and naive Bayes. The Cleveland and Starlog heart-related disease datasets were taken from the UCI collection and utilized to perform performance testing on each strategy. According to their findings, the DNN obtained 97 percent accuracy, which was greater than the other approaches [44]. The most prevalent arrhythmia that can be connected to myocardial infarction mortality in humans is called PVC. It is thought to be a prelude to more serious cardiac arrhythmias such as atrial and ventricular fibrillation [45]. The same objective was accomplished with a 96 percent accuracy rate using multi-scale Eigenspace analysis on 12-lead ECG data [46]. In [47], the MPA algorithm was used to find the best settings for CNN. The MPA-CNN ECG method for classifying and grouping can be done in the following ways:

(1) This study may be used to increase the prediction ability of models that take cardiac abnormalities into account, and it can be used for datasets that are both broad and varied.
(2) Real-time monitoring of cardiac patients is necessary for the development of effective algorithms, the extraction of features, and categorization.
(3) This study employs complex categorization methods. These models complement the MPA algorithm to create meaningful classification outputs with increased accuracy, and they have the potential to improve classification process accuracy.
(4) The findings corroborate previous research. Most existing techniques are slower and less accurate than the MPA-CNN method. MPA with CNN classifier had detection precision levels of 99.31 percent (MIT-BIH), 99.76 percent (EDB), and 99.47 percent (EDB) (INCART).

To address the limitations of traditional machine learning approaches, a new approach was developed that uses a multi-layered dilated CNN and a bidirectional RNN to identify arrhythmia signals in ECG recordings. The multi-layered dilated CNN layer is a shallow convolutional layer that extracts features from ECG data. It does this by taking the data as input. The researchers were able to achieve the degree of precision that was anticipated. Traditional machine learning no longer has to rely on feature extraction, which may take a significant amount of time and require a substantial amount of prior biological knowledge.

The heart illness arrhythmia, sometimes referred to as unstable heartbeats, is considered a major health concern. According to statistics, around 12% of the world’s population dies from this ever-growing disease or series of disorders annually [4]. It occurs when the body’s electrical activity differs from the typical process, which results in the heart beating erratically. It hinders adequate blood supply and oxygen to essential organs. The ECG is critical in diagnosing arrhythmias because it records heart rate and rhythm and is essential for electrical information on cardiac activity and associated disorders. Clinical detection of arrhythmias involves analyzing every heartbeat recorded in an individual patient’s ECG data. On the other hand, manual ECG recording analysis is time-consuming and prone to errors since ECG records are needed to document irregularly occurring arrhythmias [14]. As a result, an automated technique to aid doctors is required.

## 3. Methodology

In this research, we present an approach for detecting and classifying heart arrhythmia disease from a different dataset, which is an extremely unbalanced dataset. The suggested approach is separated into four main phases:

i. Divide the dataset into two sets: training (70%) and testing (30%).
ii. Apply cleaning techniques.
iii. Apply data augmentation techniques.
iv. Design the proposed Reseek-Arrhythmia model for detecting and classifying heart arrhythmia disease.

To train the proposed model, two different datasets, namely PTB and MIT-BIH, were utilized to detect and classify cardiac arrhythmia disease.

### 3.1. PTB Dataset

The PTB Diagnostic ECG Database (https://www.physionet.org/content/ptbdb/1.0.0/ accessed on 4 August 2023) is a publicly available dataset widely used in ECG analysis and arrhythmia detection research. This dataset provides a rich source of annotated ECG recordings from a diverse patient population with various cardiac conditions, including arrhythmia. Including recordings from healthy individuals also allows for assessing the specificity of deep learning models for arrhythmia detection. The dataset is annotated with diagnosis codes based on the Minnesota Code, which provides a standardized coding system for ECG diagnoses and facilitates the comparison of results across different studies. The dataset consists of 14,552 ECG beats, of which 4046 are classified as normal and 10,506 as abnormal.

### 3.2. MIT-BIH Dataset

The publicly available MIT-BIH dataset (https://www.physionet.org/content/mitdb/1.0.0/ accessed on 4 August 2023) is the collection of ECGs used for arrhythmia detection and classification. The MIT-BIH arrhythmia dataset comprises 109,446 ECG beats with a sampling frequency of 125 Hz, presented in a CSV file format. The dataset contains 87,554 ECG beats for training and 21,892 ECG beats for testing. Then, the dataset is classified by categorizing the ECG beats into five distinct classes: N, S, V, F, and Q. These classes are defined in Table 2, providing clear and organized information about the different arrhythmia types present in the dataset.

**Figure 3:**
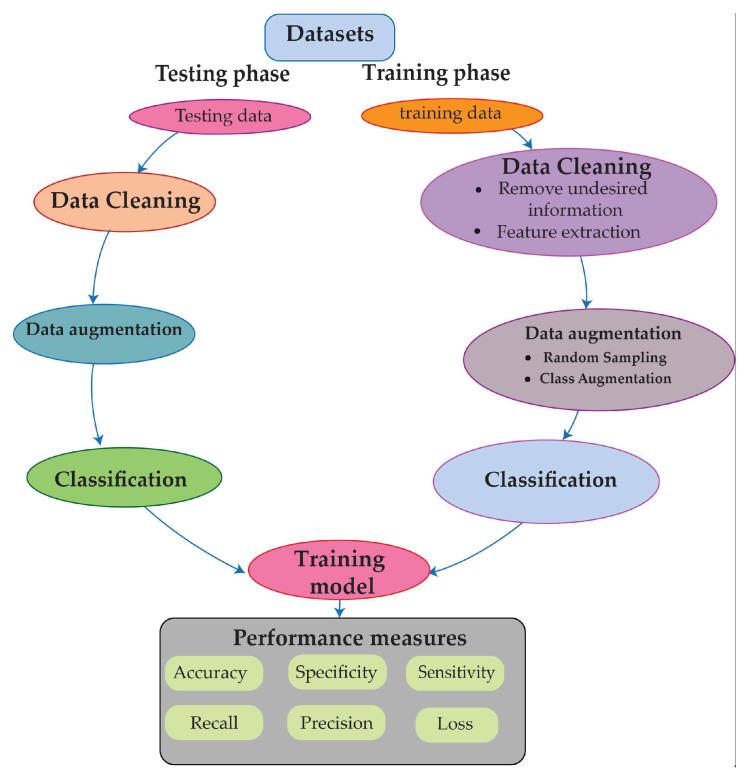
Different steps are followed in the testing and training phases.

After gathering data, they are split into training and testing segments, as shown in Figure 3. 

After separating the data, cleaning techniques were used to remove and eliminate unwanted data from the original dataset (such as duplicates, nulls, and additional information). Subsequently, to combat overfitting issues, this study used data augmentation techniques. Moreover, different techniques such as random sampling, class augmentation, and normalization were used throughout the data augmentation process. Then, a process known as min–max normalization was carried out, which rescaled the values to fall somewhere between 0 and 1. The following is the normalization formula:

(1) $$f\left(x\right)=\frac{x-x_{min}}{x_{max}-x_{min}}$$

where $x_{min}$ and $x_{max}$ stand for the lowest and highest possible values of the feature, respectively. 

The rescaling method was used to save computational resources and make the data easy to understand. Then, we finally proposed a Reseek-Arrhythmia model, which is discussed in the Section 3.3.

### 3.3. Proposed Model

**Figure 4:**
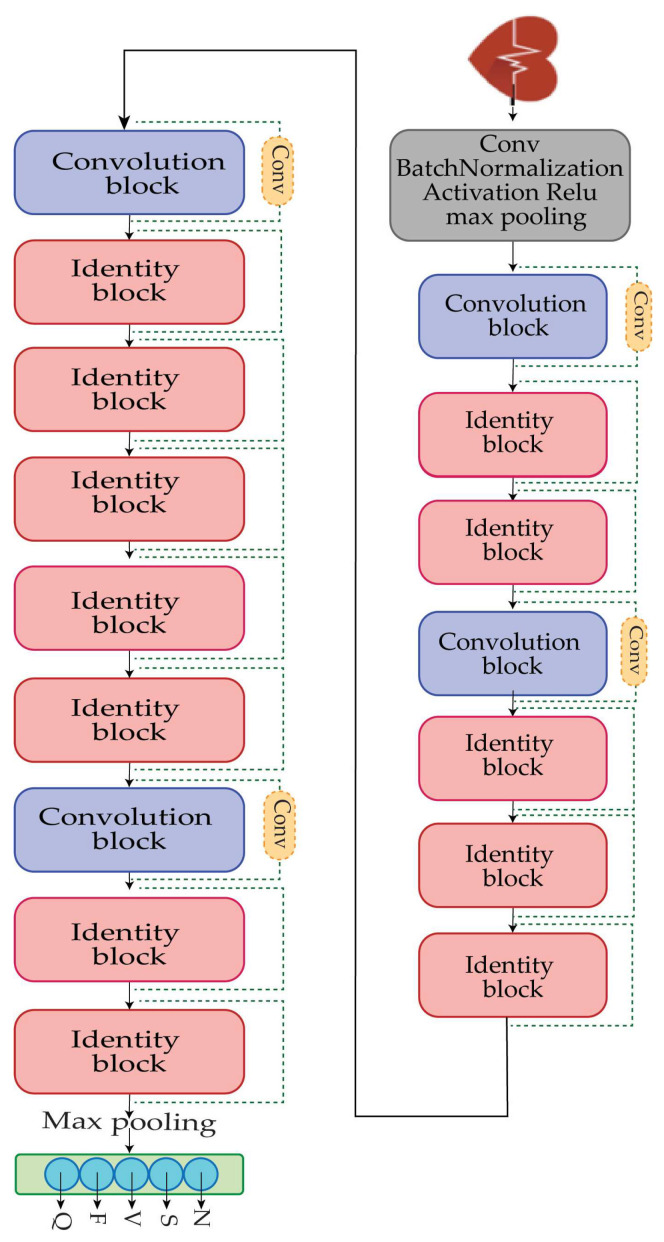
Shows the visual representation of the proposed model’s architecture, using ResNet50 as a base model. The architecture has various building blocks, skip connections, and convolutional layers.

The fundamental purpose of this study is to provide a solution for the detection and classification of heart arrhythmia disease. The dataset that is used in this study is extremely unbalanced, as shown in Table 2. For the detection and classification of heart arrhythmia disease, the Reseek-Arrhythmia model is proposed, which consists of four convolutional blocks and twelve identity blocks, along with the convolution layer, normalization layer, activation function layer, and max pooling layer, as shown in Figure 4. ResNet50 was used as the foundational framework in this study, which is a well-known deep neural network design noted for its ability to train deep networks well by tackling the vanishing gradient problem with residual blocks. Each of the convolutional blocks and identity blocks will be discussed in the Section 3.4.

The experiments were performed on Dell computers (Dell, Round Rock, TX, USA) configured with a Core i5 CPU, 8 GB of RAM, and Windows 10. The software environment and packages utilized during the development of the proposed Reseek-Arrhythmia model include:

- Python: The Python programming language was the core language used for developing the model due to its versatility and strong support for machine learning frameworks.
- TensorFlow: TensorFlow, an open-source machine learning framework developed by Google, was employed for building and training the deep learning model.
- Keras: Keras, a high-level neural network API written in Python, was used as an interface for building and configuring the neural network layers.
- NumPy: NumPy, a fundamental package for numerical computations in Python, was employed for handling mathematical operations and data manipulation.
- Pandas: The Pandas library was used for data preprocessing and manipulation, facilitating efficient handling of datasets and data frames.
- Matplotlib and Seaborn: These libraries were utilized for data visualization, aiding in the creation of various graphs and plots to present the model’s performance.
- Scikit-learn: Scikit-learn, a machine learning library for Python, provided essential tools for evaluating the model’s performance and implementing machine learning algorithms.
- Jupyter Notebook: Jupyter Notebook was used as the interactive coding environment, enabling code execution, data exploration, and result visualization in an integrated manner.

These software components and packages collectively provided a robust foundation for the development, training, and evaluation of the Reseek-Arrhythmia model. The combination of these tools facilitated efficient implementation and analysis of the deep learning techniques applied in this study. Each of the convolutional blocks and identity blocks will be discussed in the Section 3.4.

### 3.4. Convolutional Block

**Figure 5:**
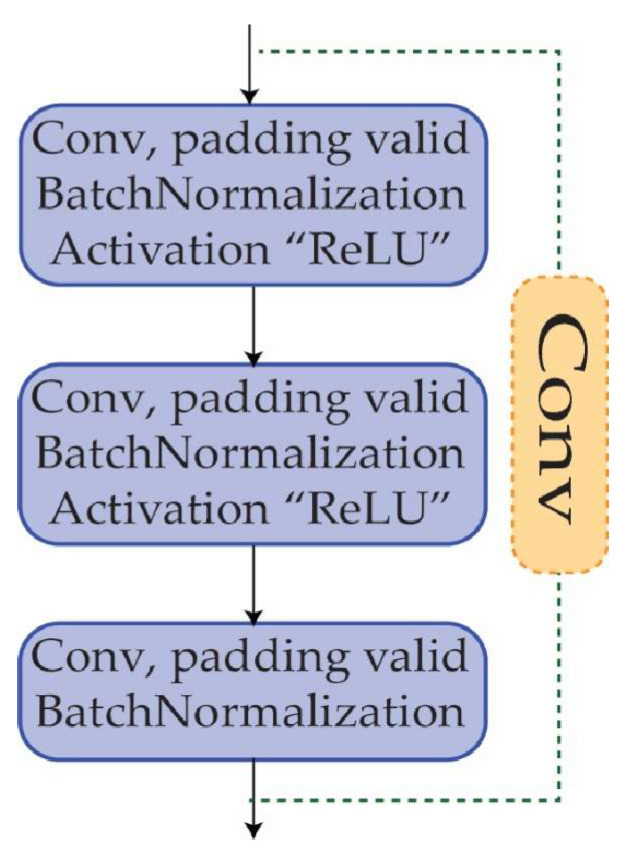
The convolutional block specifications.

Another sort of building block that is often utilized in CNNs is the convolutional block [48]. It is made up of a series of convolutional layers, batch normalization, activation layers, and, if desired, a max pooling layer. A convolutional block is implemented to extract features from the input data. The convolutional block is comprised of a number of convolutional layers, each of which is responsible for performing convolutional operations on the input data in order to identify significant patterns and characteristics. The convolutional block contributes to the neural network’s depth, allowing it to learn more complicated characteristics from the input data. The network may learn to detect more and more abstract information by stacking numerous convolutional blocks, which is necessary for effective picture identification, as shown in Figure 5.

### 3.5. Identity Block

**Figure 6:**
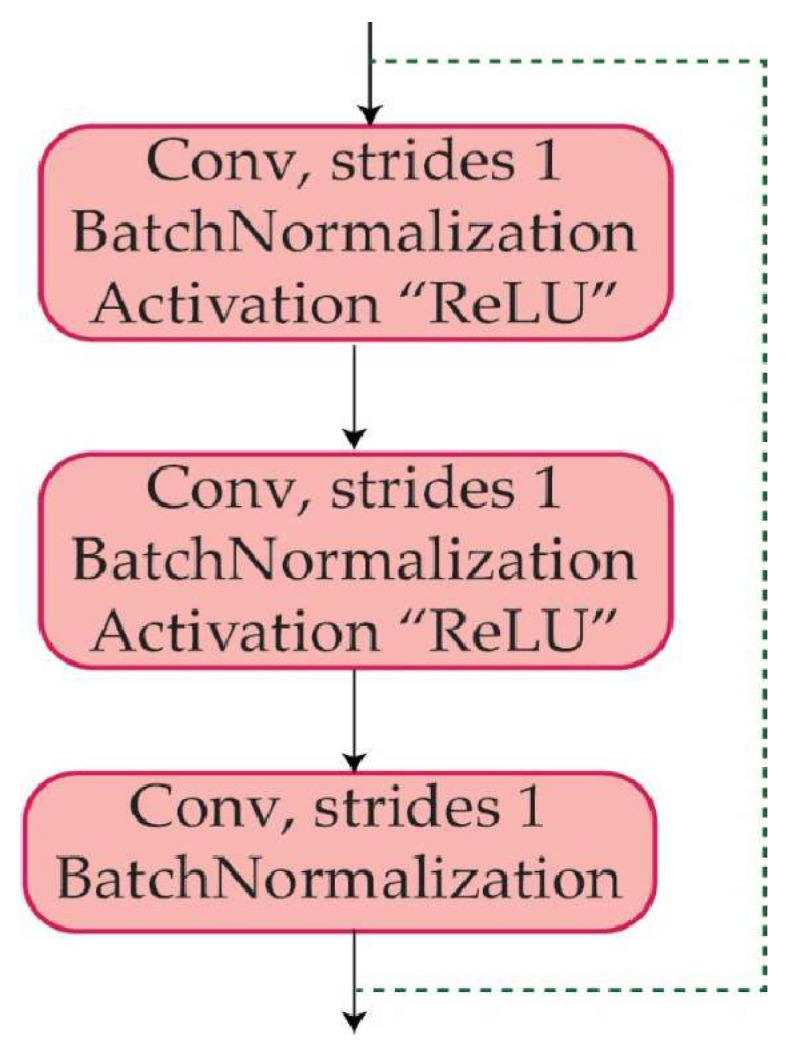
The layers used in each identity block.

An identity block is a sort of residual block that enables a neural network to bypass specific layers. The input to an identity block is appended straight to the output of the block, allowing the network to “skip over” the block if further processing of the input is not required. An identity block is made up of a series of convolutional layers, followed by batch normalization and activation layers, and finally the addition of the input and output tensors. The identity block is intended to help prevent the problems of vanishing and gradients that can arise while training deep neural networks. It enables the network to learn more efficiently by allowing direct information flow via a shortcut connection between them. The identity block improves the flow of information from one layer to the next by keeping the input and output tensors in the same dimension, where each of the identity blocks consists of multiple layers such as convolution, batch normalization, and activation layers, as can be observed in Figure 6.

## 4. Results

To evaluate the effectiveness of the proposed Reseek-Arrhythmia model in classifying arrhythmia diseases, several evaluation metrics were utilized. The Adam optimizer with a learning rate (Lr) of 0.001, beta1 of 0.9, and beta2 of 0.999 was employed to optimize the model for achieving the best possible solution. The beta1 and beta2 parameters control the decay rate of the first and second moments of the gradient and accept values between 0 and 1 [49].

Confusion metrics were used to evaluate the outcome parameters, including accuracy (ACC), recall, precision, F1-score, and loss. These metrics provide a comprehensive understanding of the model’s performance. In the context of confusion metrics, “true positives” (TP) refer to observations that are both expected and actual positives, while “false positives” (FP) occur when an observation is expected to be positive but turns out to be negative. “True negatives” (TN) are observations that are projected to be negative and turn out to be negative, and “false negatives” (FN) are instances where performance indicators are incorrectly labeled as negatives.

The proposed Reseek-Arrhythmia model incorporates various performance metrics to assess its classification capabilities. Accuracy measures the overall correctness of the model’s predictions, while precision evaluates the model’s ability to accurately identify positive instances. Recall quantifies the model’s capacity to identify all positive instances, and the F1-score combines precision and recall into a single metric. Additionally, the loss is used as a performance indicator that measures the deviation between the model’s predicted values and the actual labels. 

By employing these performance metrics, the proposed model’s effectiveness in detecting and classifying arrhythmias is thoroughly evaluated. The inclusion of these metrics enables comparisons with previous approaches and demonstrates the superior performance of the Reseek-Arrhythmia model in accurately identifying and classifying arrhythmia diseases.

### 4.1. Accuracy

The accuracy metric, denoted as *ACC* [46], is a common measure used to evaluate the performance of a model. It is calculated by dividing the total number of correctly predicted instances (true positives and true negatives) by the total number of instances, as described by Equation (2).

(2) $$ACC=\frac{TP+TN}{TP+TN+FP+FN}$$

**Figure 7:**
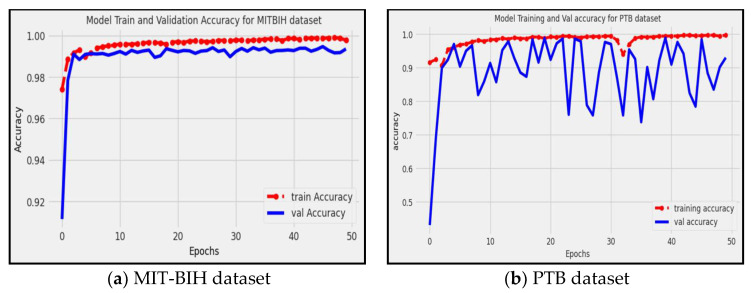
Demonstrates the model’s accuracy for the MIT-BIH and PTB datasets.

Figure 7 shows that the proposed model achieved approximately 0.9935 and 0.9350 accuracy on the MIT-BIH and PTB datasets, respectively. This indicates that the proposed model correctly predicted approximately 99.35% of the instances in the dataset, considering both true positive (*TP*) and true negative (*TN*) predictions. The red line represents the accuracy of the training data, while the blue line represents the accuracy of the validation data. Accuracy provides an overall measure of how well the model performs in classifying instances correctly, and it has been proven that the proposed model is able to successfully identify and classify arrhythmia patterns with a high level of accuracy.

### 4.2. Specificity

Specificity [13] is a statistical metric that measures the model’s ability to accurately predict true negatives for each category in consideration. It is also referred to as the true negative rate, which can be calculated by dividing true negatives by the sum of true negatives and false positives, as shown in Equation (3).

(3) $$SPE=\frac{TN}{TN+FP}$$

**Figure 8:**
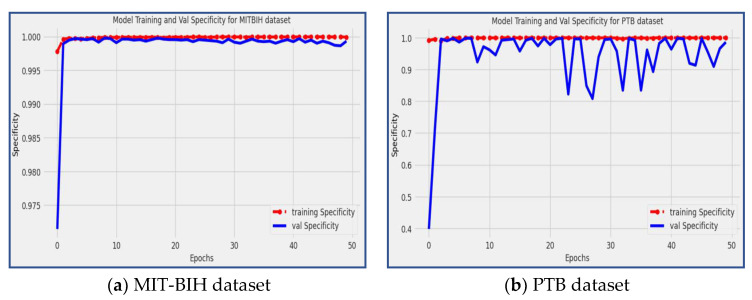
The model training and validation specificity for the MIT-BIH and PTB datasets.

Figure 8 showcases the specificity scores achieved by the proposed model during the training and validation phases on the PTB and MIT-BIH datasets. The red line corresponds to the specificity of the training data, while the blue line represents the specificity of the validation data. From the figure, it can be observed that the proposed model achieved a specificity score of 98.59% on the PTB dataset and an impressive specificity score of 99.93% on the MIT-BIH dataset. Specificity measures the model’s ability to correctly identify true negative (*TN*) instances, indicating how well it distinguishes normal, non-arrhythmic patterns. The high specificity scores achieved by the model demonstrate its proficiency in accurately classifying non-arrhythmic instances, further solidifying its effectiveness in arrhythmia detection and classification tasks.

### 4.3. Sensitivity 

Sensitivity [3], also known as the true positive rate, is a statistical measure that evaluates how effectively a model can identify individuals with a specific ailment or disease. To calculate sensitivity, the number of true positive cases is divided by the sum of true positives and false negatives, as shown in Equation (4).

(4) $$SEN=\frac{TP}{TP+FN}$$

**Figure 9:**
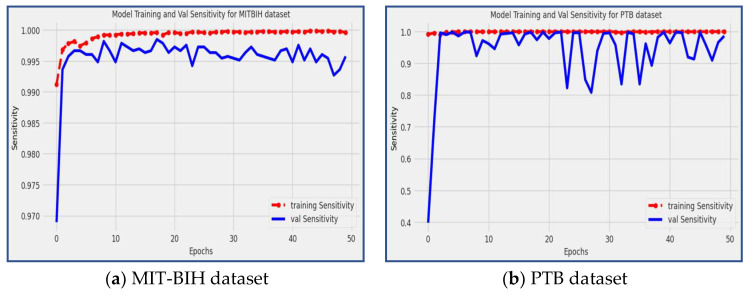
The model training and validation sensitivity for the MIT-BIH and PTB datasets.

The proposed model demonstrated remarkable sensitivity scores on both the MIT-BIH and PTB datasets, achieving 98.59% and 99.55%, respectively. Figure 9 visually represents the performance of the model during the training and validation phases. The red line in the figure represents the sensitivity scores during model training, while the blue line corresponds to the sensitivity scores during validation. Sensitivity measures the model’s ability to correctly identify true positive (*TP*) instances, indicating its effectiveness in detecting and classifying arrhythmic patterns. The high sensitivity scores attained by the model indicate its strong ability to accurately identify instances of arrhythmias, making it a valuable tool for the early detection and classification of cardiac arrhythmia disease.

### 4.4. Precision

Precision [8] is another valuable metric for evaluating the accuracy of a classification model, especially when dealing with imbalanced datasets. Precision measures the effectiveness and accuracy of a model in correctly classifying true positive (*TP*) cases among all positive predictions. Precision is calculated by dividing the number of *TP* predictions by the sum of *TP* and *FP* predictions, as shown in Equation (5):

(5) $$Precision=\frac{TP}{TP+FN}$$

**Figure 10:**
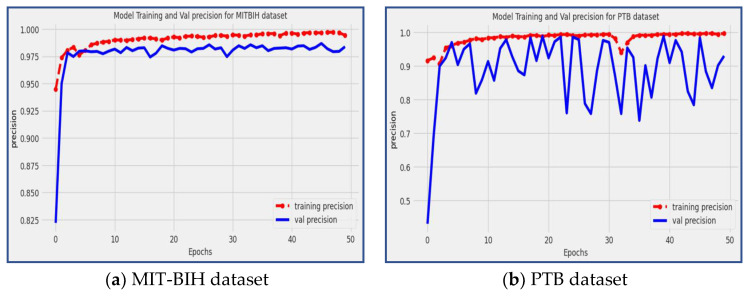
The precision of the suggested model during training and validation using MIT-BIH and PTB datasets.

Precision is a crucial metric that provides valuable insights into the model’s accuracy in identifying specific types of arrhythmias. A high precision score, close to 1, indicates that the model has a low rate of false positive predictions, meaning that it accurately classifies positive instances with a high level of precision. A low accuracy score closer to 0 indicates that the model has a larger rate of false positives, which indicates a less accurate categorization for positive cases. The proposed model achieved precision of 98.39% and 92.97% on the MIT-BIH and PTB datasets, respectively, as shown in Figure 10. The precision scores reflect the model’s ability to accurately detect and classify particular types of arrhythmias, making it an accurate tool for the precise identification and categorization of cardiac arrhythmia conditions.

### 4.5. Recall

On the other hand, recall measures the model’s effectiveness in correctly identifying positive cases out of all the actual positive cases in the dataset. In the context of evaluating a deep CNN model’s ability to recognize positive classifications in real-world scenarios, the recall metric (also known as sensitivity) is employed. The recall value ranges from 0 to 1, representing the model’s ability to identify positive instances. A higher value means better performance in recognizing true positives. The recall is calculated by dividing the number of true positive predictions by the sum of true positive predictions and false negative (*FN*) predictions, as shown in Equation (6):

(6) $$Recall=\frac{TP}{TP+FN}$$

Recall, also known as sensitivity, is another crucial metric for evaluating the performance of the proposed model. It measures the model’s ability to correctly identify positive instances out of all the actual positive instances present in the dataset. A high recall score, close to 1, indicates that the model has a low rate of false negative predictions, meaning it effectively captures and classifies positive instances with a high level of sensitivity.

**Figure 11:**
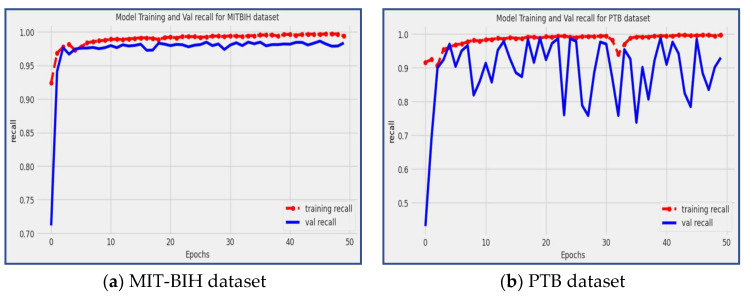
The model training and validation recall for the MIT-BIH and PBT datasets.

In our proposed model, impressive recall scores of 98.39% and 92.97% were achieved on the MIT-BIH and PTB datasets, respectively, as illustrated in Figure 11. The recall result shows the model’s capacity to recognize and categorize arrhythmia patterns properly, particularly in situations when the timely identification and effective treatment of cardiac arrhythmias are essential for patient wellbeing.

### 4.6. Loss

Loss is a critical metric in machine learning that measures how well the model’s predictions align with the actual target values during the training process. A lower loss value indicates that the model’s predictions are closer to the true target values, signifying a better fit of the model to the training data.

**Figure 12:**
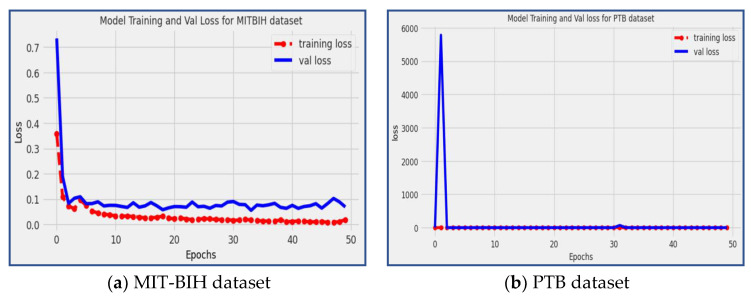
Loss of the suggested model during training and validation using MIT-BIH and PTB datasets.

In our proposed model, we achieved a loss of 0.688 on the MIT-BIH dataset and 0.2564 on the PTB dataset, as depicted in Figure 12. The red line in the figure represents the training loss, while the blue line represents the validation loss.

The observed low loss values indicate that the proposed model has effectively learned and captured patterns in the training data and can generalize well to unseen data (validation data). By achieving low loss values on both datasets, the proposed model demonstrates its ability to efficiently identify and classify heart arrhythmia disease patterns with high accuracy, precision, recall, and minimal errors. 

## 5. Discussion

This study addresses the challenges associated with manually analyzing electrocardiogram (ECG) records, which include time-consuming procedures and potential inaccuracies due to irregularly occurring arrhythmias. The focus of the article is on developing an automated method using deep learning techniques to assist physicians in identifying and categorizing arrhythmia disorders. The suggested methodology consists of four major steps. Firstly, split the dataset into training and testing sets, which allows the model to learn patterns and make predictions on unseen data. Next, cleaning techniques are applied to remove any unwanted data that may hinder the performance of the model. Data augmentation techniques are then used to combat overfitting, which is a phenomenon where the model becomes too specialized for the training data and performs poorly on new data.

Preprocessing techniques are applied to make the data more manageable. This includes rescaling the data using min–max normalization, which transforms the values into a specific range to ensure consistent processing. The proposed model for this study is called the Reseek-Arrhythmia model. It is designed to handle the highly imbalanced dataset used in the study, meaning that certain arrhythmia types may have significantly fewer examples compared to others. The Reseek model is made up of the convolution block and the identity block, where the convolutional blocks have the responsibility of extracting features from the input data that are necessary for analyzing the patterns and attributes of various arrhythmias. In order to overcome the issue of vanishing gradients, which can impede learning in DNNs, the identity blocks enable information to flow across short-cut connections.

To assess the efficiency of the proposed approach, two publicly accessible datasets are utilized: the PTB Diagnostic ECG Database and the MIT-BIH dataset. The MIT-BIH dataset contains a significant number of ECG beats categorized into five arrhythmia classes: N, S, V, F, and Q, whereas the PTB dataset is classified into two classes: normal and abnormal. The suggested model’s performance is measured using a variety of parameters, including accuracy, sensitivity, specificity, precision, recall, and loss. Accuracy assesses the model’s overall accuracy of predictions, whereas precision and recall assess the model’s capacity to properly identify positive cases and cover all relevant examples, respectively. Loss is a statistic that measures the difference between the model’s predicted value and the actual value. This study intends to give insights into the usefulness of the suggested automated method for recognizing and categorizing arrhythmias using DL techniques by analyzing the model’s performance using these parameters. 

## 6. Conclusions

The study’s outcome emphasizes the relevance of arrhythmia as a cardiovascular disease that affects a huge population worldwide. Early detection and accurate diagnosis of arrhythmias are crucial for effective therapy and management of the condition. Deep learning strategies have gained popularity in recent years for their potential in the early identification and classification of arrhythmias. This study focuses on utilizing electrocardiogram (ECG) data to develop practical deep-learning approaches and create an efficient model for classifying cardiovascular illnesses. The proposed architecture in this study combines different convolutional blocks and identity blocks, employing the MIT-BIH and PTB datasets. These convolutional blocks analyze various features and patterns present in the data, enabling the model to learn the distinctive characteristics associated with different arrhythmias. On the other hand, identity blocks are employed to address the problem of vanishing gradients, which can occur during the training of deep neural networks. The identity blocks establish shortcut connections that allow direct information flow between layers, mitigating the vanishing gradient issue. This facilitates more efficient learning and enhances the overall performance of the model. The study reports impressive results after training the proposed model using the combined datasets. The proposed model outperformed with an accuracy of 99.35% and 93.50%, showing that it properly diagnosed arrhythmias in the majority of instances. The model also obtained a low loss value of 0.688 and 0.2564, which assesses the difference between predicted and categorized actual values. The proposed model’s efficacy demonstrates its potential to aid healthcare practitioners in the early diagnosis and classification of arrhythmias.

## Figures and Tables

**Table 1:** Systematic literature review.

S.No	Ref. No	Detection Techniques	Accuracy (%)
1	Atrial Fibrillation Detection Based on a Residual CNN Using BCG Signals (2022) [3]	CNN	96.8
2	An automated detection of heart arrhythmias using machine learning technique: SVM (2021) [11]	SVM	95.92
3	ECG signal classification with binarized convolutional neural network (2020) [12]	BNN	86.8
4	Arrhythmia Classifier using Binarized Convolutional Neural Network for Resource-Constrained Devices (2022) [13]	BCNN	96.45
5	Arrhythmia classification with ECG signals based on the optimization-enabled deep convolutional neural network (2020) [4]	CNN	93.19
6	Optimal multi-stage arrhythmia classification approach (2020) [14]	Extreme gradient boosting tree	97
7	Low-power ECG arrhythmia detection SoC with STT-MRAM and LDMAC unit (2021) [15]	STT-MRAM	85.1
8	Classification of Obstructive Sleep Apnoea from single-lead ECG signals using convolutional neural and Long Short Term Memory networks (2021) [5]	CNN, LSTM	90.92
9	Cardiac arrhythmia detection using deep learning (2017) [6]	DCNN	92
10	Multiresolution wavelet transform-based feature extraction and ECG classification to detect cardiac abnormalities (2017) [16]	SVM	98.9
11	High-performance personalized heartbeat classification model for long-term ECG signal (2017) [17]	GRNN	88
12	A new personalized ECG signal classification algorithm using block-based neural network and particle swarm optimiza-tion (2016) [18]	BBNN	97
13	An approach for ECG beats classification using adaptive neuro-fuzzy inference system (2016) [19]	ANFIS	96
14	An automated ECG beat classification system using deep neural networks with an unsupervised feature extraction technique (2019) [20]	DL	99.73
15	Arrhythmic heartbeat classification using ensemble of random forest and support vector machine algorithm (2021) [21]	SVM, RF	98.21
16	Electrocardiogram soft computing using hybrid deep learning CNN-ELM (2020) [22]	CNN + EML	97.50
17	ECG beat classification using PCA, LDA, ICA and discrete wavelet transform (2013) [23]	SVM	99.28
18	Application of higher-order cumulant features for cardiac health diagnosis using ECG signals (2013) [24]	NN, LS-SVM	94.52
19	Cardiac arrhythmia prediction using improved multilayer perceptron neural network (2013) [25]	MLPNN	95.1
20	DWT-based feature extraction from ECG signal (2013) [26]	MLPNN	85
21	Heartbeat classification using particle swarm optimization (2013) [27]	BMLPNN	76
22	Artificial neural network models based cardiac arrhythmia disease diagnosis from ECG signal data (2012) [28]	MNN-generalized FFNN	86.67
23	An effective ECG arrhythmia classification algorithm (2011) [29]	PNN	99.71
24	ECG beat classification using features extracted from teager energy functions in time and frequency domains (2011) [30]	NN	95
25	Classification of cardiac arrhythmia using WT, HRV, and fuzzy c-means clustering (2011) [31]	FCM-HRV	99.05
26	Arrhythmia detection based on morphological and time-frequency features of t-wave in electrocardiogram (2011) [32]	MLP, ANN	96.7
27	Classification of arrhythmia in heartbeat detection using deep learning (2021) [33]	CNN + LSTM + Attention	99.29

**Table 2:** The different classes of the MIT-BIH arrhythmia dataset.

S/No.	Class	Beats Type	Number of Beats
1	N	Normal beat	90,589
2	S	Supraventricular premature beat	8039
3	V	Premature ventricular contraction	7236
4	F	Fusion of ventricular and normal beat	2779
5	Q	Unclassifiable beat	803
Total number of beats in the MIT-BIH dataset for training			109,446

## Data Availability

Data will be available upon suitable request to the corresponding author.
